# Impact of the Hereditary P301L Mutation on the Correlated Conformational Dynamics of Human Tau Protein Revealed by the Paramagnetic Relaxation Enhancement NMR Experiments

**DOI:** 10.3390/ijms21113920

**Published:** 2020-05-30

**Authors:** Ryosuke Kawasaki, Shin-ichi Tate

**Affiliations:** 1 Department of Mathematical and Life Sciences, Graduate School of Science, Hiroshima University, 1-3-1 Kagamiyama, Higashi-Hiroshima, Hiroshima 739-8526, Japan; ryosuke-kawasaki@hiroshima-u.ac.jp; 2 Department of Mathematical and Life Sciences, Graduate School of the Integrated Sciences for Life, Hiroshima University, 1-3-1 Kagamiyama, Higashi-Hiroshima, Hiroshima 739-8526, Japan; 3 Research Center for the Mathematics on Chromatin Live Dynamics (RcMcD), Hiroshima University, 1-3-1, Kagamiyama, Higashi-Hiroshima, Hiroshima 739-8526, Japan

**Keywords:** tau protein, intrinsically disordered protein, NMR, paramagnetic relaxation enhancement

## Abstract

Tau forms intracellular insoluble aggregates as a neuropathological hallmark of Alzheimer’s disease. Tau is largely unstructured, which complicates the characterization of the tau aggregation process. Recent studies have demonstrated that tau samples two distinct conformational ensembles, each of which contains the soluble and aggregation-prone states of tau. A shift to populate the aggregation-prone ensemble may promote tau fibrillization. However, the mechanism of this ensemble transition remains elusive. In this study, we explored the conformational dynamics of a tau fragment by using paramagnetic relaxation enhancement (PRE) and interference (PRI) NMR experiments. The PRE correlation map showed that tau is composed of segments consisting of residues in correlated motions. Intriguingly, residues forming the β-structures in the heparin-induced tau filament coincide with residues in these segments, suggesting that each segment behaves as a structural unit in fibrillization. PRI data demonstrated that the P301L mutation exclusively alters the transiently formed tau structures by changing the short- and long-range correlated motions among residues. The transient conformations of P301L tau expose the amyloid motif PHF6 to promote tau self-aggregation. We propose the correlated motions among residues within tau determine the population sizes of the conformational ensembles, and perturbing the correlated motions populates the aggregation-prone form.

## 1. Introduction

Neurofibrillary inclusions are the hallmark of neurogenerative diseases such as Alzheimer’s disease (AD) as the most common of the tauopathies [1,2]. These inclusions within neuronal cells consist of neurofibrillary tangles (NFTs) [3,4,5]. The filamentous aggregates, NFTs, contain the microtubule-associated protein tau self-assembled to paired helical filaments (PHFs) [6]. The progression of tau aggregates correlates with the severity of dementia and neurodegeneration in AD [7,8]. Disease-associated hereditary mutations increase tau NFT deposition and thus promote pathologies [9,10,11]. In vitro and in vivo experiments have shown that mutations in tau enhance PHF aggregation [12]. Exploring the molecular mechanism of tau self-aggregation and why the hereditary mutants promote tau PHF formation should aid the development of therapeutics against tauopathies.

**Figure 1:**
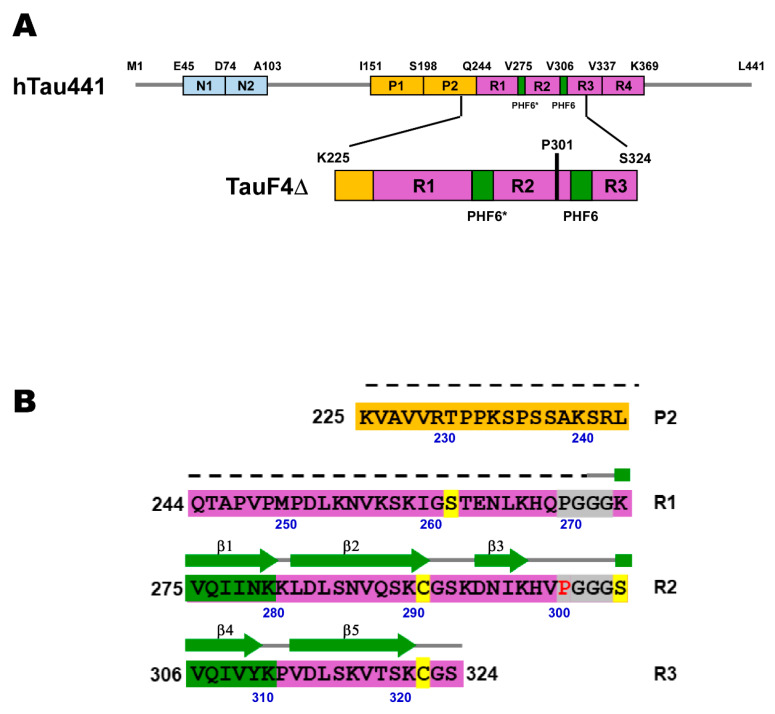
(**A**) The domain architecture of the longest isoform of the human tau protein, which comprises 441 residues (hTau441), and the domain architecture of the fragment (TauF4Δ) used in this work. N1 and N2 are acidic regions (cyan), P1 and P2 are proline-rich regions (yellow). R1, R2, R3 and R4 are the pseudo-repeats (purple) that engage in microtubule binding and constitute the core of tau filament structures. The two hexapeptides highlighted in green are essential for tau fibrillization, which are named PHF6* and PHF6. (**B**) The primary sequence of TauF4Δ. The secondary structures found in the heparin-induced tau filament structure are indicated on the primary sequence and the dotted line indicates the unstructured part in the tau filament [19]. Two PGGG sequences near the amyloid motifs (PHF6* and PHF6 in green) are highlighted in gray, in which P301 is marked in red. The residues in yellow are the positions used for MTSL spin-labeling.

The longest isoform of human tau is 441 residues in length [13] (Figure 1A). Human tau is largely disordered and a representative of the intrinsically disordered protein (IDP) family [14,15]. Tau has four microtubule-binding (MTB) repeats, R1 to R4, which span residues 243–365 (Figure 1A). The tau MTB repeats are not random coils but contain residual structures [15,16,17], and the parts harboring the residual structures constitute the core of PHFs formed in vivo and also in polyanion-induced PHF assembly in vitro [18,19].

Two hexapeptide regions at the beginning of R2 and R3 crucially engage in PHF aggregation, named PHF6* (^275^VQIINK^280^) and PHF6 (^306^VQIVYK^311^) [9,20,21,22,23] (Figure 1B). The isolated hexapeptides per se polymerize [9,24]. The isolated PHF6* and PHF6 motifs are partially populated in β-structures, and the sparsely populated β-structures function as seeds in forming the cross-β structure in tau PHFs [20,25].

Tau isolated as a recombinant protein from bacteria is highly soluble and does not spontaneously aggregate under physiological concentrations, and can be stored in solution over a long period [4,26,27,28]. Tau is, therefore, an intrinsically highly soluble protein. The initiation of tau aggregation in vitro requires polyanions, such as heparin [4,12]. Tau switches from a highly soluble state to an aggregation-prone form upon exposure to external stimuli.

Hereditary mutations in tau that are linked to frontotemporal dementia and Parkinsonism linked to chromosome 17 (FTDP-17) promote neurofibrillary deposits [10,13]. Many of the FTDP-17 tau mutants raise aggregation-prone properties [29,30]. One representative FTDP-17 mutation, P301L, is located adjacent to the PHF6 motif (Figure 1B), which promotes self-aggregation by increasing the β-structure propensity within the MTB repeat region [31,32]. The FTDP-17 mutants provide clues for understanding the molecular mechanism that initiates tau aggregation.

Solution NMR experiments have shown that the P301L mutation changes the local structure of R2 but has a negligible effect on the secondary structure propensity of the entire soluble monomeric tau [17]. NMR has not observed aggregation-prone conformations of tau because these conformational ensembles are too sparsely populated to be detected [17,33].

Other biophysical approaches, including cross-linking mass spectrometry, hydrogen/deuterium (H/D)-exchange mass spectrometry, and double electron-electron resonance (DEER) have consistently suggested that monomeric tau adopts two distinct conformational ensembles in solution [23,34,35,36]. One ensemble is inert to fibrillization, whereas the other ensemble is aggregation-prone and promotes fibrillization [23,34,35]. Tau molecules in a soluble ensemble appear to adopt compact conformations where the PHF6* and PHF6 sequences are shielded through intramolecular contacts with other parts in tau [23,34,35]. In contrast, tau molecules in the aggregation-prone ensemble populate extended conformations that expose the PHF6* and PHF6 motifs, thereby promoting tau–tau interactions [23,34,35]. Triggers including phosphorylation, heparin-binding, and FTDP-17 mutations may shift the equilibrium to the aggregation-prone conformational ensemble to promote self-aggregation and subsequent fibrillization [23,35].

Correlated motions or collective motions in a protein cause residues to fluctuate in a concerted manner [37]. Collective motions in various folded proteins have been demonstrated [37], and some of these collective motions have functional roles [37,38,39]. Correlated motions have been characterized through the conformation trajectory of a target protein, and this trajectory can consist of a large number of conformations generated by molecular dynamics (MD) simulations [37]. Applying the same MD simulation approach to the largely unstructured tau protein is challenging. This is because tau samples a much larger conformational space when compared with a folded protein, which is difficult to sample fully in a reasonable simulation period. The difficulty in applying the MD approach to tau prohibits the analysis of correlated motions in tau.

Recently, an established NMR experiment with paramagnetic spin labeling detected correlated motions among residues in an unstructured protein [40]. The experiment relies on the enhanced ^1^H transverse relaxation rate that arises when the paramagnetic unpaired electron spin comes in spatial proximity to the proton under observation, causing paramagnetic relaxation enhancement (PRE) [40,41,42]. Kurzbach and coworkers reported that residue-specific correlation among the PRE data collected from proteins with spin-labels at different positions enables mapping of residues undergoing correlated motions [40,43]. The PRE map provides short- and long-range correlations among residues of the protein undergoing conformational dynamics [43,44].

Kurzbach and coworkers also published an advanced PRE application to capture transient conformations of a protein labeled with two unpaired electron spins, which uses PRE interference from two different paramagnetic electron spins (paramagnetic relaxation interference, PRI) [40]. PRI only occurs when two electron spins in the protein are in close spatial proximity [43]. PRI, therefore, exclusively probes conformations that transiently occur because of conformational dynamics [40]. As in the PRE map, inter-residue PRI correlations identify dynamically correlated residues in transiently folded conformations, i.e., the PRI map [40,44]. Thus, PRE probes the major conformational ensemble, whereas PRI detects transient conformations that are much smaller in population size [45,46].

In the present work, we analyzed the conformational dynamics of the tau fragment (TauF4Δ, residues 225–324) composed of the short proline-rich sequence, R1, R2, and part of the R3 (Figure 1A). Residues of TauF4Δ constitute the core structure of the heparin-induced tau PHF [19]. We explored changes in correlated motions caused by the hereditary pathogenic mutation with PRE and PRI experiments in combination with nuclear spin relaxation and secondary structure population analyses by NMR.

The results revealed that tau consists of segments that undergo correlated motions. Notably, residues in the β-structures of the heparin-induced tau filament are coincident with those in the segments displaying correlated motions [19]. The results suggest that tau has segmental conformational dynamics, and that each segment converts to a structural element in the tau filament. In addition to the correlated motions among residues within the segments, there are also long-range correlations among distal segments. The long-range inter-segment correlations may determine the overall conformational dynamics of tau in solution.

The P301L mutation caused changes in the PRI data but did not change the PRE data noticeably. These observations indicate that the P301L mutation has limited structural impact on transient conformations while conformations in the major ensemble remain mostly unchanged. The PRI-based structural model showed that the P301L mutation induces aggregation-prone forms in a low population ensemble, which exposes the amyloid motif PHF6. The sparsely populated P301L mutant structure modeled from PRI data explains its elevated aggregation propensity. The P301L mutation changes the transiently folded conformation by changing the mode of inter-residue correlated motions.

## 2. Results

### 2.1. P301L Mutation Effects on the Backbone Dynamics of TauF4Δ

**Figure 2:**
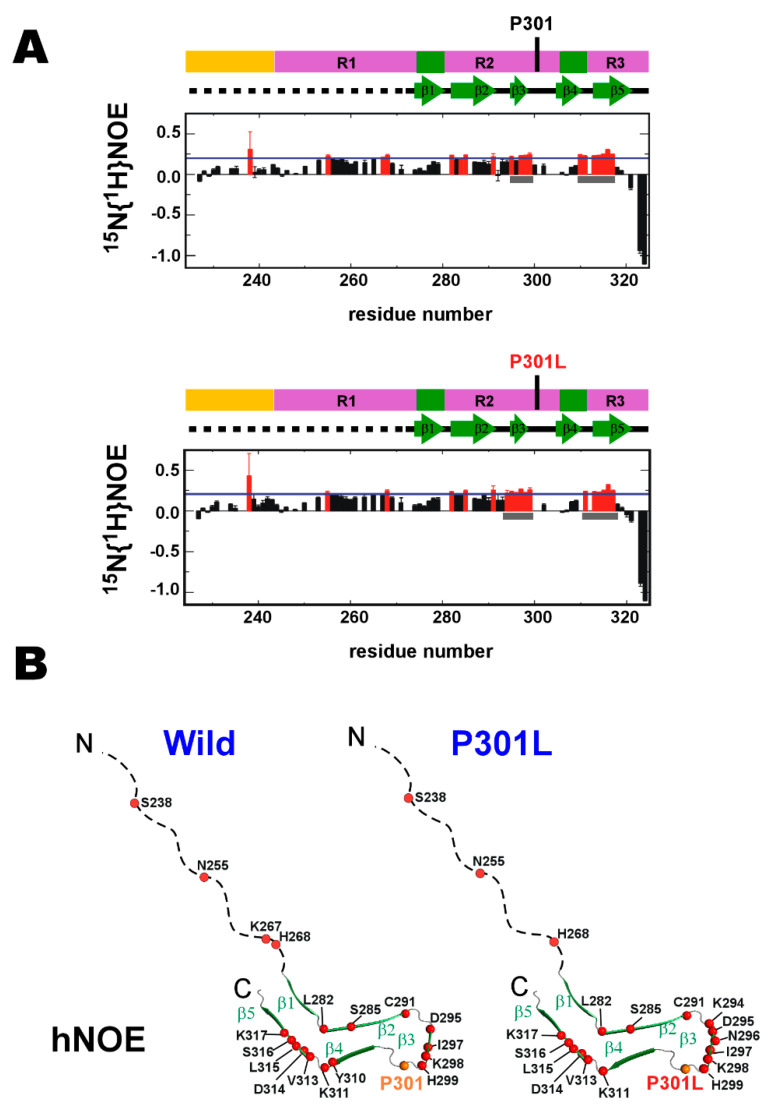

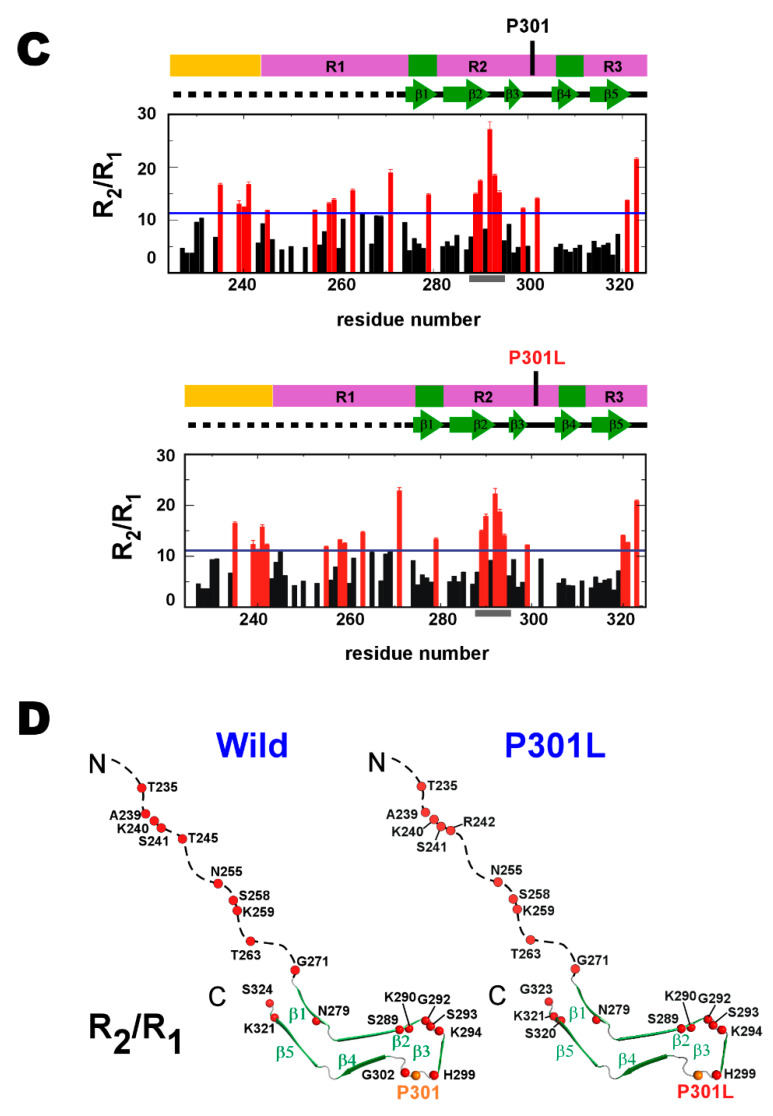
Comparing the conformational dynamic parameters between the wild-type and P301L mutant TauF4Δ. (**A**) Heteronuclear ^1^H-^15^N nuclear Overhauser effects (hNOEs) are plotted. The upper and lower panels are the wild-type and P301L mutant TauF4Δ.The domain architecture of tau and the secondary structures formed in the heparin-induced tau filament are drawn on each plot. hNOE values that are larger than the average value (blue line) are marked in red. The gray bars indicate the positions for β3 and β5. (**B**) Residues with hNOE values greater than the average are marked by red circles on the heparin-induced tau filament structure (PDB ID: 6QJH) [19]. (**C**) *R*_2_/*R*_1_ values; the wild-type (upper) and the P301L mutant (lower). *R*_2_/*R*_1_ values shown in red are higher than the average value (blue line). Gray bars mark the position for the linker connecting β2 and β3. (**D**) Residues with *R*_2_/*R*_1_ values above the average are marked by red circles on the tau filament structure.

The P301L mutation caused only marginal changes to hNOE values (Figure 2A), suggesting P301L mutation has a limited impact on the backbone dynamics on the nsec timescale. Intriguingly, residues in the wild-type TauF4Δ with higher hNOEs than the average value are clustering in β3 and β5 of the heparin-induced tau filament structure (PDB ID: 6QJH, ‘snake filament’) [19] (Figure 2B). This was also the case for the P301L mutant TauF4Δ (Figure 2B). The residues in the β3 and β5 regions appear to have a similar level of structural order in both the wild-type and P301L mutant (Figure 2A, gray bars).

The *R*_2_/*R*_1_ plot for residues in the wild-type TauF4Δsuggests that the segment linking the parts corresponding to β2 and β3 has apparent conformation dynamics on the ms timescale, as evident by the larger *R*_2_/*R*_1_ values for residues in this segment [47,48] (Figure 2C, gray bar). The P301L mutant also has higher *R*_2_/*R*_1_ values for residues in the same segment as the wild-type protein (Figure 2C, gray bar). The distribution of the residues showing the higher *R*_2_/*R*_1_ values over the average in the P301L mutant is close to that in the wild-type, demonstrating P301L mutation does not change the backbone dynamics on the msec timescale (Figure 2D).

The correlation between the spectral densities *J*_eff_(0) and *J*(ω_N_) for the wild-type and P301L mutant TauF4Δ confirms the segment linkingβ2 and β3 has significantly greater fluctuations on the ms timescale [49,50] (Figure S1). 

Notably, the residues corresponding to the β-structures and those for the unstructured parts in heparin-induced tau filament have different dynamic properties (Figure 2B,D). The monomeric soluble tau fragment is entirely dynamic in conformation (Figure 2A), but the conformation dynamics are not uniform for all the residues. Instead, the tau fragment retains the segmental structures discriminated by their different dynamics properties (Figure 2B,D). The P301L mutation caused no apparent changes in this segmental conformation dynamics (Figure 2B,D).

We further characterized the P301L associated changes in conformational dynamics by reduced spectral density analysis, which describes the conformation dynamics on different time scales measured at the Larmor frequencies of 0, *ω*_N_ (71.0 MHz), and *ω*_h_, which approximates *ω*_H_ (700.3 MHz) [51,52,53]. The data showed that the P301L mutation did not induce any clustered changes in the spectral densities (Figure S2). However, the P301L mutation did affect the dynamics of all residues in TauF4Δ (Figure S2), indicating that this mutation affected residue dynamics distal from the mutation site and that the dynamics of residues are interconnected. Thus, there should be correlated motions that link these distal residues in tau to the mutation site. 

### 2.2. P301L Reduces the Transiently Populated β-Conformation of R2 

**Figure 3:**
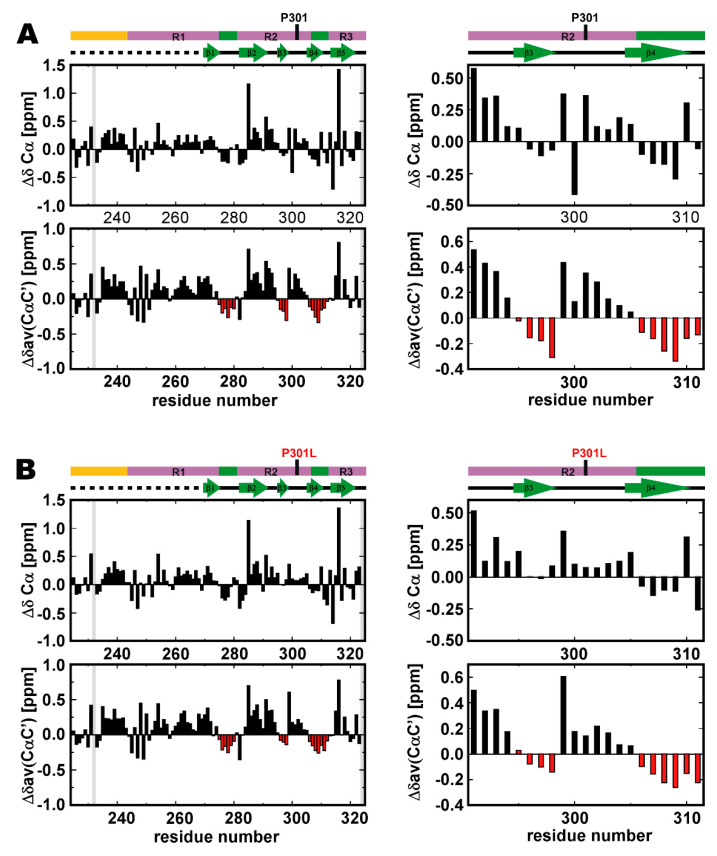
Secondary chemical shifts of TauF4Δ. (**A**) Secondary chemical shifts for ^13^C_α_, ΔδC_α_ and the averaged secondary chemical shifts for ^13^C_α_ and ^13^C’, Δδ_av_(C_α_C’), for the wild-type are shown in the left panels. The expanded plots around the mutation site, P301, are placed in the right panels. The averaged secondary chemical shits are calculated as [3ΔδC_α_+ 4ΔδC’]/7 [25]. Regions showing β-structure propensity are identified by negative Δδ_av_(C_α_C’) values that extend over several residues and are marked in red. (**B**) The secondary chemical shifts ΔδC_α_ and Δδ_av_(C_α_C’) for the P301L mutant are presented. The gray bar represents the residue did not show a resolved signal.

Protein backbone NMR chemical shifts, in particular ^13^Cα and carbonyl ^13^C’ nuclei, are sensitive probes for elucidating the secondary structure population [54,55,56,57,58,59]. Three segments comprising consecutive residues (more than three residues) gave negative ^13^Cα secondary chemical shifts (Δδ^13^Cα) in the wild-type TauF4Δ (Figure 3A), showing that these segments are nascent β-strand structures [56]. These segments are PHF6*, PHF6 and the region comprising the residues 296–298, which region corresponds to β3 in the heparin-induced filament [19] (Figure 1B). The average secondary shifts for ^13^Cα and carbonyl ^13^C’, Δδ_av_(^13^Cα^13^C’) = [3Δδ^13^Cα) + 4Δδ^13^C’]/7, provided a stronger indication of the secondary structure propensities [25], which confirmed that the above three segments have high β-structure propensities (Figure 3A). Previous work has revealed that the amyloid motifs, PHF6* and PHF6, have high β-structure propensities, but did not focus on the β-structure propensity of the segment corresponding to β3 [15,25].

The positive Δδ_av_(^13^Cα^13^C’) values for the ^301^PGGG^304^ sequence in wild-type tau indicates that the PGGG motif has the propensity to form a β-turn [25]. The other PGGG sequence locates upstream of PHF6* (Figure 1B). Residues in the sequence ^270^PGGG^273^ also give positive Δδ_av_(^13^Cα^13^C’) values, and thus also display a propensity to form a β-turn (Figure 3A).

The P301L mutation reduced the magnitudes of Δδ_av_(^13^Cα^13^C’) values for the segment corresponding to β3 in the tau filament, suggesting that this segment has a reduced propensity to form a β-structure in the P301L mutant (Figure 3B).

The P301L mutation also reduced the magnitude of Δδ_av_(^13^Cα^13^C’) for the ^301^PGGG^304^ sequence (Figure 3B). The change in the secondary chemical shifts suggests the P301L mutation destabilizes the β-turn of ^301^PGGG^304^. Residues following the ^301^PGGG^304^ sequence showed no apparent changes in secondary chemical shifts, Δδ^13^Cα and Δδ_av_(^13^Cα^13^C’). The P301L mutation does not change the β-structure propensity of the PHF6 motif (Figure 3B). This observation is consistent with previous work [15,25]. 

Taken together, the P301L mutation destabilizes the β-structure of β3 in the tau filament and the β-turn of ^301^PGGG^304^. The P301L mutation should increase the flexibility of residues 295–305 in TauF4Δ, leading this region of the protein to preferentially adopt an extended conformation. 

### 2.3. Correlated Motions Among Residues in TauF4Δ

PREs can reveal transient contacts among distal residues in IDPs [41,42,60,61]. Paramagnetism from an unpaired electron-spin enhances the transverse relaxation rate of the amide proton (^1^H^N^) in a protein, and the rate (PRE rate) is denoted by ^1^H^N^-Γ_2_. Enhancement in the relaxation rate depends on the average distance between the ^1^H^N^ and the unpaired electron spin (X), and also the effective reorientation time of the amide proton-electron (^1^H^N^-X) vector [41,42]. 

The vast conformational space of an IDP sample enables an unpaired electron labeled at a specific site to transiently contact with various protons in the protein. If a segment of residues in the protein fluctuates cooperatively, the PRE effects on those residues should change in a correlated manner. PRE data collected for a set of labeled tau mutants, where each tau in the set has an electron spin at the different position, will give a correlation map for ^1^H^N^-Γ_2_ values (PRE rates), which enables the identification of residues undergoing cooperative motion [40,44]. 

A PRE correlation map provides quantitative relations between PRE rates of a pair of residues, which come from the multiple PRE data collected for the proteins harboring a spin-label at different positions. Four TauF4Δ mutants were used with spin-labels at residues 262, 291, 305, and 322 (Figure 1B). All the PRE data for the wild-type and the P301L mutant TauF4Δ are provided in Figures S3 and S4.

**Figure 4:**
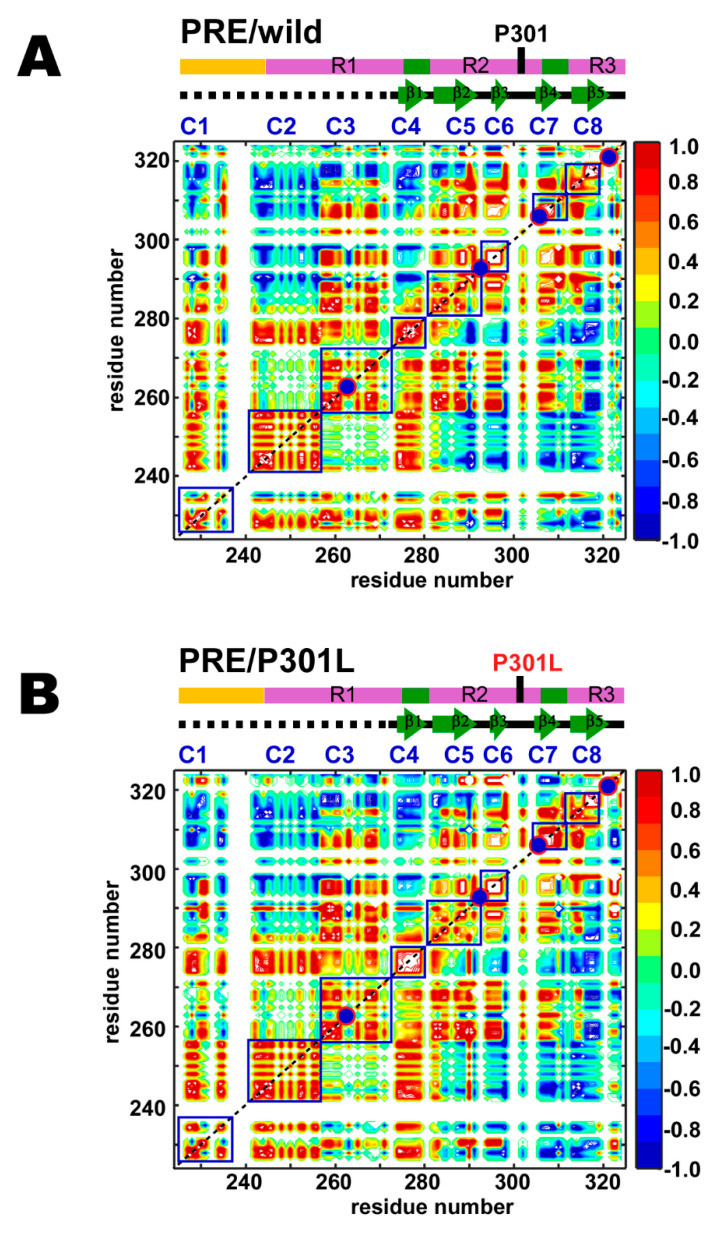
PRE correlation maps for the (**A**) wild-type and (**B**) P301L mutant of TauF4Δ. Colors indicate correlated (red), anti-correlated (blue) and uncorrelated (green) contours. Only residue positions with observable PRE rates for two or more mutants are given. MTSL labeling positions are shown on the maps by the red circles filled in dark blue. The segments with locally correlated residues are marked by the blue squares labeled C1–C8.

The PRE correlation maps for the wild-type and P301L mutant TauF4Δ were compared (Figure 4A,B). Positive correlations (*corr*_i,j_, Equation (1)) are shown in red, non-correlated regions in green, and anti-correlated regions in blue: Residues showing a positive correlation (red) fluctuate concertedly in reference to the spin-label, while residues showing a negative correlation (blue) move in an anti-correlated fashion.

It should be noted that the correlation maps are drawn in contour lines without filling the colors inside to demonstrate the difference in the contour structures among the correlating residues, as exemplified by the different patterns inside the C4 regions in the wild-type and the P301L mutant (Figure 4). Therefore, the area comprising the maximal (+1.0) or the minimal (–1.0) correlations shows flat top or bottom colored in white, as typically shown in the C6 regions in the wild-type and the P301L mutant (Figure 4), while the white area surrounded by the red lines in C6 region comprises +1 correlations.

The PRE map for the wild-type TauF4Δ demonstrates that the tau fragment has eight locally correlated segments, as marked by the blue squares on the map (Figure 4A). The sequential consecutive residues within each segment show positive correlations. Notably, segments ranging from C4 to C8 correspond to the β-structures in the heparin-induced tau filament (Figure 4A). Residues in collective motion may promote β-structure formation during fibrillization.

Some residues in the proline-rich region (PRR) of the wild-type TauF4Δ show correlated motion (C1) (Figure 4A). The C1 segment contains a microtubule-binding site in tau [15]. The C1 segment has a regulatory role in tau function as phosphorylation of T231 and S235 in this segment reduces tau binding to microtubules and prohibits tubulin polymerization into microtubules [62,63,64,65,66].

The R1 contains two segments that undergo correlated motion (C2 and C3) (Figure 4A). Tau has four major microtubule-binding sites [15,25]. Residues 245–255 in C2 represent a microtubule-binding site [15]. Besides the abovementioned microtubule-binding sites, the other two sites are ^275^VQIINKKLDLSNV^287^ and ^306^VQIVYKPVDLSKV^318^ [15]. Residues 275–287 cover segments C4 and C5 (Figure 4A), in which the C4 segment corresponds to the PHF6* motif (^275^VQIINK^280^). The other binding site (residues 306–318) includes segments C7 and C8. The C7 segment contains the PHF6 motif (^306^VQIVYK^311^).

As described above, segments that have correlated residues constitute functionally relevant regions, including microtubule-binding sites, an aggregation seed and β-structure scaffolding cross-β in fibrils. This finding suggests that each segment with correlated residues behaves as a functional unit in a similar fashion to a domain in folded proteins.

The P301L mutation changes the correlated motion among the sequentially neighboring residues in the segments C4, C5, and C7 (Figure 4B). The degree of the correlation among the residues in the C4 segment for the P301L mutant increases; the area showing maximal correlation (+1) colored in white inside the contour lines is expanded in the P301L mutant on the plot (Figure 4B). Residues in the C5 segment of the P301L mutant gain long-range correlations within this segment, as shown by the elongated antidiagonal correlations crossing over the diagonal when compared with that of the wild-type (Figure 4B). The C7 segment in the P301L mutant expanded the correlations among residues (Figure 4B). The anti-correlation streak in the map limits the correlations among the residues within the C7 segment of the wild-type (Figure 4A), while the corresponding correlations become positive in the C7 segment of the P301L mutant (Figure 4B).

The wild-type and P301L mutant TauF4Δ showed similar long-range PRE correlations among segments but show small but significant differences in some parts of the correlation maps (Figure 4). The segment C1 in the wild-type correlates to all other segments from C2 to C8 (Figure 4A). The C1 segment in the P301L mutant has different correlations among residues within the segment, which further altered long-range inter-segment correlations to the segments from C2 to C8 (Figure 4B). In particular, the P301L mutant has lost many of the positive correlations and thus enhanced the negative correlations from C1 to C7 and C8 segments (Figure 4B). The changes in the long-range correlations suggest that the C-terminal region comprising the C7 and C8 segments in the P301L mutant moves in an opposite direction to the corresponding motion in the wild-type. 

Intriguingly, the segments C2 and C3 in the wild-type show no correlations in motion, indicating that these two segments behave independently (Figure 4A). C2 is anti-correlated to the region comprising C5, C6 and C7, as evident by their correlations primarily in blue, whereas C3 has positive correlations to these segments, which give mainly red correlations. The long-range inter-segment correlations from C2 and C3 do not change in the P301L mutant (Figure 4B). 

The PRE map revealed that TauF4Δ has intimate inter-relating correlated motions that regulate the conformational dynamics of the entire fragment. The result emphasizes that TauF4Δ is not just a random coil of uniform flexibility, but samples conformations preferentially that are formed by the inter-correlated structural dynamics. Tau has been reported to adopt an ‘S-shape’ or ‘paper clip-like’ fold, in which the N- and C-terminal regions transiently contact to the MTB repeat domains [67,68,69]. By taking into account previous observations, the long-range positive inter-segment correlations found for the C1 segment in the wild-type TauF4Δ suggest that the C1 segment folds over the MTB repeat domains (R1, R2 and part of R3) to adopt the ‘S-shape’ or ‘paper clip-like’ fold (Figure 4). The compact global fold of tau should embrace the amyloid motifs PHF6* and PHF6 within the structure to prevent tau aggregation [21,23,36,70,71].

The anti-correlated relation of C1 to C7 and C8 segments in the P301L mutant suggests that the P301L mutant TauF4Δ destabilizes the transient ‘S-shape’ or ‘paper clip-like’ fold by shifting the C7 and C8 segments away from the C1 segment. The change in conformational dynamics may be caused by the reduced β-turn propensity of the ^301^LGGG^304^ sequence in the P301L mutant. The anti-correlated motion for the region comprising the C7 and C8 segments in the P301L mutant exposes PHF6, which explains the elevated aggregation propensity of the P301L mutant [29].

### 2.4. PRI Data Reveals Conformation Changes Caused by P301L are Exclusively Associated with Transient Structures

The PRI maps for TauF4Δ harboring two paramagnetic spin-labels at different sequence positions showed apparent differences between the wild-type and the P301L mutant. We used two pairs of spin-labeling, residues 262–305 and 291–322 (Figures S3 and S4). PRI probes conformational ensembles that differ from those detected by PRE [40,43,44]. PRI exclusively detects transiently formed compact structures of tau, in which two electron spins approach to be in close proximity [43].

**Figure 5:**
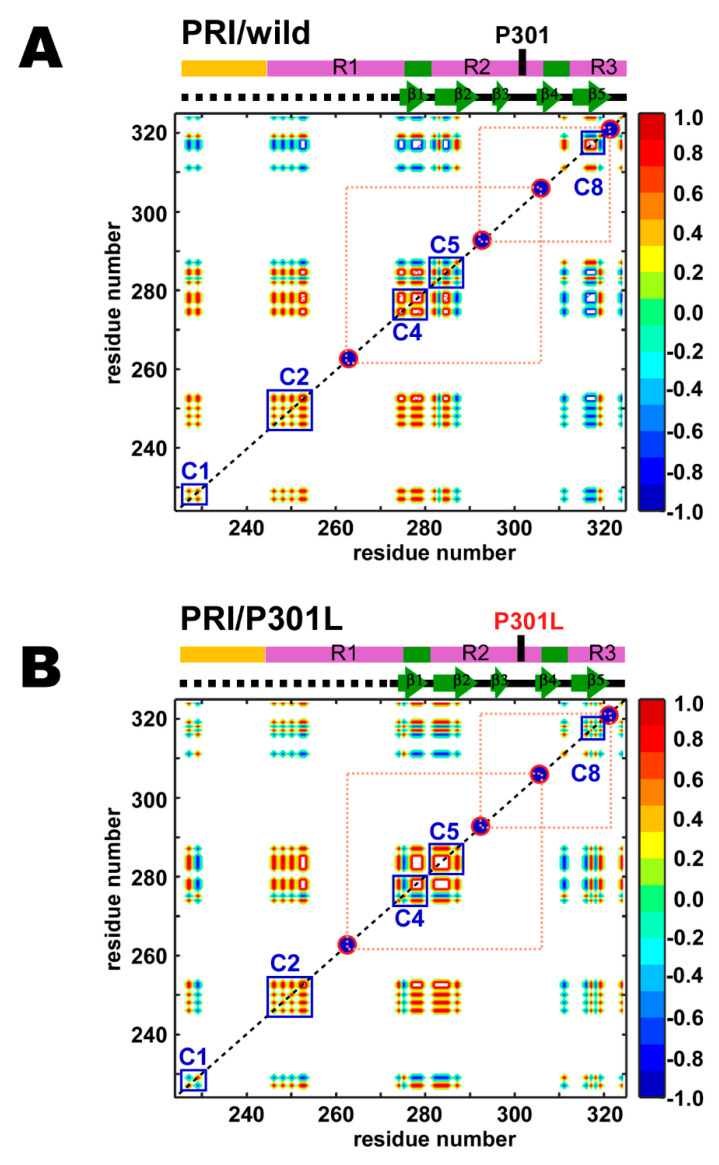
PRI correlation maps for the (**A**) wild-type and (**B**) P301L mutant of TauF4Δ. Only residues with observable PRI rates for two variants are shown. The red circles filled in dark blue indicate the positions of the MTSL labels. The MTSL positions connected by orange dotted lines are used for simultaneous spin-labeling. Segments comprising residues with local correlations are marked by the blue squares labeled by the closely corresponding segments in the PRE map. Colors of the contours indicate the degrees of correlations as used in the PRE maps: correlated (red), anti-correlated (blue) and uncorrelated (green).

The PRI effect becomes apparent when two unpaired electrons, X(1) and X(2), are in spatial proximity (< 50 Å), under which conditions X(1)-^1^H^N^ and X(2)-^1^H^N^ dipoles interfere with each other [43]. The magnitude of the PRI rate (Equation (2)) depends on the term <3cos^2^*θ* – 1>, where *θ* is the angle between X(1)-^1^H^N^ and X(2)-^1^H^N^ vectors, and the angle bracket indicates the ensemble average. Thus, the PRI depicts conformational ensembles exclusively in compact forms with orientations between X(1)-^1^H^N^ and X(2)-^1^H^N^ vectors [43]. When the average projection angle *θ* approaches 0° or 180° in compact forms and two unpaired electrons are spatially proximate, the PRI rate becomes positive. In contrast, if the conformations populated give an average projection angle *θ* close to 90°, the PRI rates are negative. The PRI map provides correlations among residues of the substructures that differ to those obtained in the PRE map [44]. We used two different pairs of spin-labeling for TauF4Δ, and these pairs are defined in Figure 5 as the dotted lines. The PRI map gave five correlated segments out of the eight segments observed in the PRE map (Figure 5).

The P301L mutation changed the PRI correlations among residues within the C1, C4, C5 and C8 segments (Figure 5A,B). In the C4 segment of the wild-type, the C-terminal residues of the PHF6* motif correlates with its N-terminal residues (Figure 5A). However, the corresponding correlation is diminished by the streak of anti-correlation in the C4 segment of the P301L mutant (Figure 5B). 

The P301L mutant showed a positive correlation among residues within the C5 segment, and these residues further correlated with the C-terminal residues in the C4 segment (Figure 5B). The wild-type does not have the corresponding correlation among the residues in the C5 segment (Figure 5A).

The P301L mutant loses the correlation among residues within the C8 segment (Figure 5B), while the residues in the C8 segment of the wild-type are positively correlated (Figure 5A). The loss of the correlation among the residues in the C8 segment of the P301L mutant disrupts the inter-segment correlation between C1 and C8 segments (Figure 5B), which was negatively correlated in the wild-type (Figure 5A). Additionally, the correlation among the residues within the C1 segment of the P301L mutant changed negative (Figure 5B) from a positive correlation in the wild-type (Figure 5A). Transient compact structures of the P301L mutant appear to have a flexible C8 segment that has no apparent correlations to other parts in TauF4Δ, whereas the wild-type displays anti-correlated motion for the corresponding segments (Figure 5). The P301L mutant appears to adopt different conformations from those of the wild-type in the transiently folded ensemble.

### 2.5. Structural Changes in the Transient Compact Structures of the P301L Mutant TauF4Δ 

**Figure 6:**
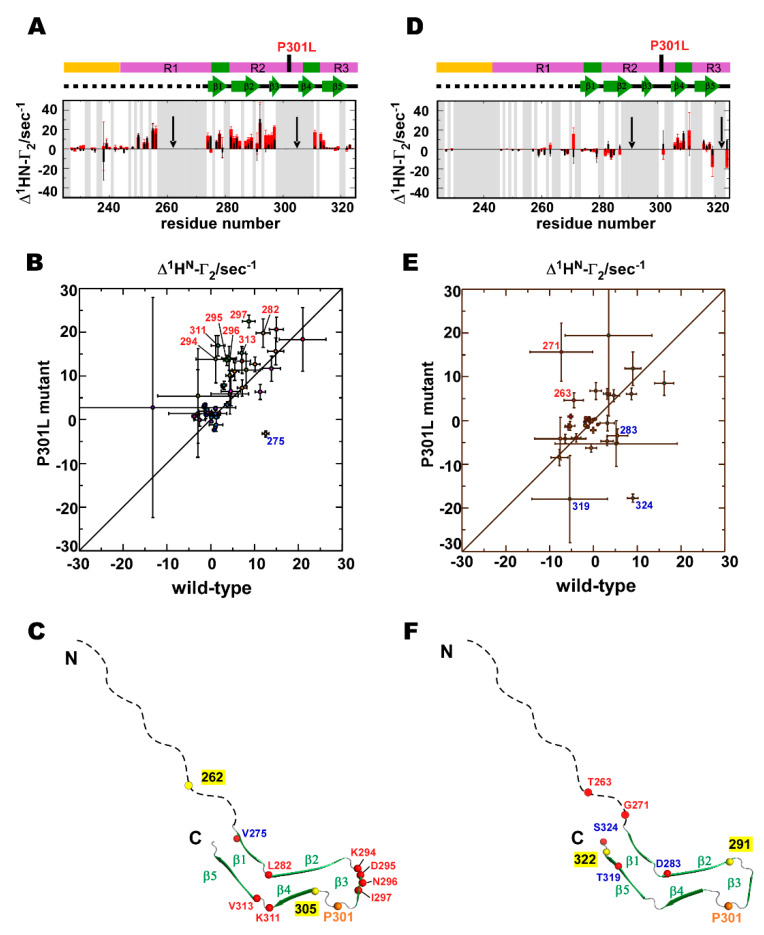
Comparison of PRI rates (Δ^1^H^N^-Γ_2_) between the wild-type and P301L mutant TauF4Δ. (**A**) PRI rates for the wild-type (black bars) and P301L mutant (red bars) TauF4Δ labeled at residues 262 and 305. The black arrows indicate the positions of the MTSL labels. (**B**) The correlations of the PRI rates between the wild-type and P31L mutant labeled at 262 and 305 are mapped. Residues showing significant differences in rates between the two proteins are labeled. Labeling in red shows that the PRI rates have increased in the P301L mutant when compared with that of the wild-type, whereas labeling in blue indicates that the PRI rate decreased in the mutant when compared with that of the wild-type protein. (**C**) Residues having significantly changed PRI rates are indicated by the red circles on the heparin-induced tau filament structure (PDB ID: 6QJH) [19]. The colors of the residue numbers represent the same meanings as used in the PRI rate correlations. The yellow circles indicate residues that are MTSL labeled. (**D**) The PRI rates for the wild-type and P301L mutant TauF4Δ labeled at residues 291 and 322. (**E**) The PRI rate correlations between the wild-type and the P301L mutant labeled at 291 and 322. (**F**) Residues with significant changes in PRI rates in the P301L mutant are shown. Gray bars represent the residues that did not show the resolved signals on the spectra.

The difference in Δ^1^H^N^-Γ_2_ between the wild-type and P301L mutant provides semi-quantitative views on conformational changes to the transient TauF4Δ structures. We modeled plausible conformational changes to TauF4Δ caused by the P301L mutation based on changes in Δ^1^H^N^-Γ_2_ (Figure 6).

The P301L mutant with spin-labels at 262 and 305 significantly changed the PRI rates for some residues when compared with those of the wild-type (Figure 6A,B). It is remarkable that residues with significantly increased PRI rates are clustered near β3 in the heparin-induced tau filament (Figure 6C). Other residues with increased PRI rates, including L282, K311, and V313, are located at the edge of the β-strands in the tau fibril structure (Figure 6C). One residue at the edge of β1 had a reduced PRI rate in the P301L mutant (Figure 6C).

The P301L mutant with spin-labels at 291 and 322 showed substantial changes in PRI rates for five residues when compared with that of the wild-type. Residues 271 and 263 had increased PRI rates, whereas residues 283, 319, and 324 had reduced PRI rates relative to the corresponding rates in the wild-type protein (Figure 6D,E). Residues with significant changes in PRI rates are located in the unstructured region and the edge of the β-structures in the heparin-induced tau fibril (Figure 6F).

**Figure 7:**
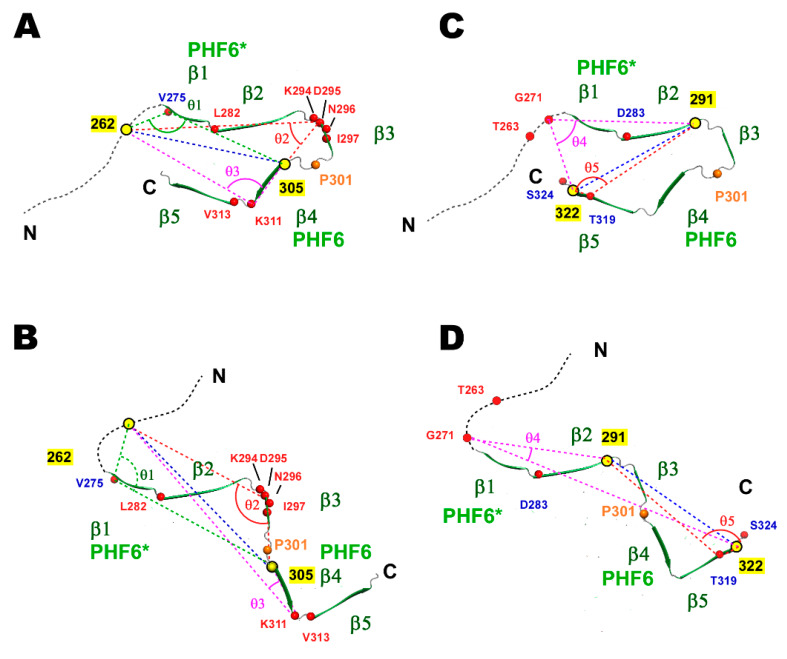
Model structures of TauF4Δ based on significant changes in the PRI rates between the wild-type and P301L mutant. Three and two representative residues are selected from TauF4Δ labeled at 262 and 305 and TauF4Δ labeled at 291 and 322, respectively, all of which showed significant changes in PRI rates for the P301L mutant. The structures are modeled from the heparin-induced tau filament structure (PDB ID: 6QJH) [19] to fulfill the presumed angle changes (*θ*_1_–*θ*_5_) suggested by the changes in the PRI rates. (**A**) A representative form of the wild-type TauF4Δ modeled based on the PRI rates for V275, N296 and K311 in the tau fragment with labeling at 262 and 305, and (**B**) also on the PRI rates for G271 and T319 in the tau fragment with labeling at 291 and 322. (**C**) A representative conformation similarly modeled based on the same set of data collected from the tau fragment labeled at residues 262 and 305, and (**D**) also for the tau fragment with labeling at 291 and 322 (**D**).

Based on the observed PRI changes for particular residues in the P301L mutant, we manually modeled the structures of the wild-type and P301L mutant using the heparin-induced tau filament structure (PDB ID: 6QJH) [19] (Figure 7).

The PRI rate for V275 in TauF4Δ labeled at 262 and 305 shifted from a positive value for the wild-type protein to a negative value for the P301L mutant (Figure 6B), implying that the inner angle *θ*_3_ between X(262)-^1^H^N^(275) and X(305)-^1^H^N^(275) approaches 180° in the wild-type and is close to 90° in the P301L mutant (Figure 7A,C). The PRI rate for D295 changed from about 5 sec^–1^ (wild-type) to 12 sec^–1^ (P301L) (Figure 6B), indicating the inner angle *θ*_1_ was close to 180° in the P301L mutant (Figure 7C). The PRI rate for K311 changed from near zero (wild-type) to about 15 sec^–1^ for the mutant. The inner angle *θ*_2_, therefore, changed from near the magic angle (54.7°) to around zero degrees (Figure 7C).

For TauF4Δ spin labeled at 291 and 322, the PRI rate for G271 changed from a small negative value (–7 sec^–1^) to a large positive value (15 sec^–1^) (Figure 6E), indicating that the inner angle *θ*_5_ changed from near 90° to a value close to 0° (Figure 7B,D). The PRI rate of T319 change from –6 sec^–1^ to –19 sec^–1^ (Figure 6E). This change suggests the inner angle *θ*_4_ approaches 90° in the P301L mutant, while the angle should be greater than 90° in wild-type tau (Figure 7B,D).

In the above discussion, the average distances between the two unpaired electrons are assumed to be close in the wild-type and P301L mutant. We think this assumption is valid because the wild-type and the P301L mutant TauF4Δ showed similar SAXS profiles in the low-q region (q < 0.1 Å), suggesting the wild-type and the mutant have similar hydrodynamic radii [72] (Figure S5). Thus, the proteins have similar molecular hydrodynamic radii, even though both proteins undergo extensive conformational dynamics.

The changes in the PRI rates observed for the proteins labeled with two spin-labels at different positions gave consistent structural models for the wild-type and mutant (Figure 7). Each structure represents the most probable form in the ensemble of structures for the wild-type and P301L mutant TauF4Δ. The P301L mutant TauF4Δ shifts the conformational ensemble, in which the closed forms burying the PHF6 sequence within the ensemble are transformed to open forms that expose the aggregation-prone sequence (Figure 7). This conformational shift is consistent with previous results [17,23,25].

## 3. Discussion

This work aimed to explore how the P301L mutation changes the conformation ensembles of Tau4Δ. Here, we summarize the conformational dynamics revealed for the wild-type and P301L mutant TauF4Δ.

(1) Residues in the wild-type TauF4Δ corresponding to β3 and β5 in the heparin-induced tau filament are slightly more ordered in terms of ns dynamics when compared with other residues (Figure 2A). The amyloid motifs, PHF6* and PHF6, are less ordered than other residues (Figure 2A). The P301L mutation does not change conformation dynamics on the ns timescale.

(2) Residues 292–294 that link the β2 and β3 segments fluctuate significantly on the μs timescale (Figure 2C). This region may function as a hinge to generate a broad range of conformations that enable various orientations between the β2 and β3 segments. The P301L mutation does not change the hinge dynamics.

(3) The amyloid motifs PHF6* and PHF6 in the wild-type TauF4Δ show higher β-structure propensities, as revealed by the secondary chemical shifts analysis (Figure 3A), which is consistent with a previous report [15]. The part corresponding to β3 in the heparin-induced tau filament also shows β-structure propensity (Figure 3A). The amyloid motifs are less ordered on the ns time scale but they show similar levels of β-structure propensities to that of the β3 part, while the amyloid motifs form β-structures more dynamically than that of the β3 part.

The P301L mutation exclusively reduced the β-structure propensity of the β3 part (Figure 3B). The result suggests that the P301L mutation destabilizes the transient folding β-structure of the β3 part while not affecting the dynamic β-structures of PHF6* and PHF6 (Figure 3B). The reduced Δδ_av_(CαC’) values for the P301L mutation indicate a reduction in the β-turn propensity of ^301^PGGG^304^ (Figure 3B). The mutated sequence ^301^LGGG^304^ has a higher preference to adopt a random coil state and thus preferentially forms an extended conformation.

(4) The wild-type TauF4Δ comprises segments of sequentially neighboring residues with correlated motion. Intriguingly, the β-structures in the heparin-induced tau filament are coincident in their positions with those segments displaying correlated motion (Figure 4A). There are also long-range correlations among segments that display local correlated motions (Figure 4A). The P301L mutation caused small but clear changes in local and long-range correlations (Figure 4B).

(5) PRI detects subsets of conformations that are lowly populated [40,44]. The PRI maps showed that the P301L mutation disturbed local and long-range correlations (Figure 5). This observation is in stark contrast to the results that the mutation had minimal impact on the PRE map (Figure 4). The data demonstrate that the P301L mutation exclusively affects transient forms of TauF4Δ, while the major forms of the P301L mutant remain mostly unchanged from those of the wild-type.

**Figure 8:**
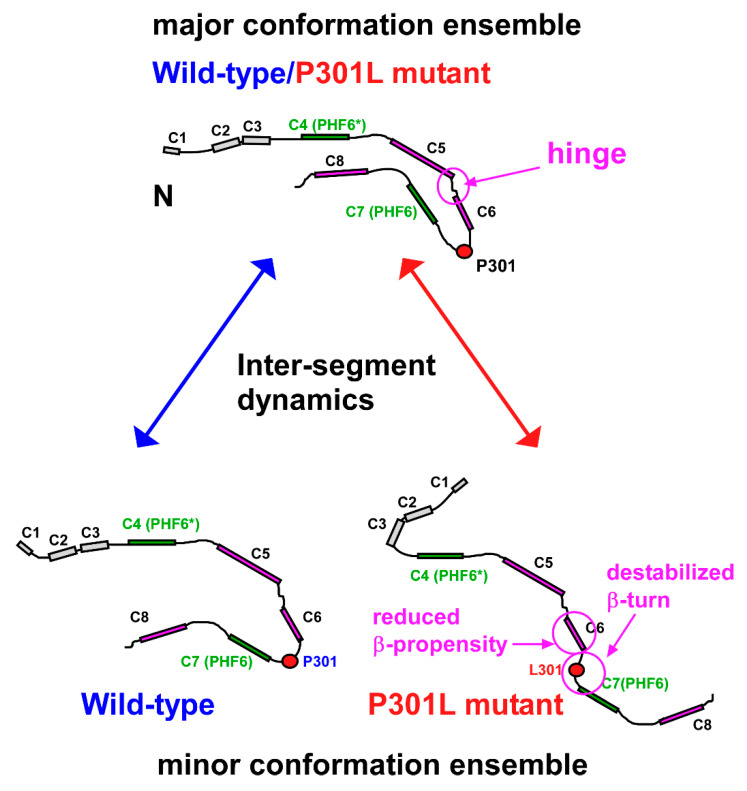
TauF4Δ segmental conformation dynamics determine the ensemble structures. The boxes represent segments that comprise residues that are undergoing correlated motions, which were identified on the PRE maps (Figure 4). Each label of the box indicates the corresponding segment name on the PRE maps. Residues in the segments C4–C8 closely overlap with residues in the β-structures (β1–β5). Segments corresponding to the amyloid motifs, PHF6* and PHF6, are green and other segments are purple. The conformations in the major ensembles of the wild-type and P301L mutant TauF4Δ should resemble each other, as indicated by the similar PRE maps (Figure 4). The P301L mutation destabilizes the β-turn structure of ^301^PGGG^304^ causing this region to adopt an extended structure preferentially. The conformational dynamics of the hinge linking C5 and C6 segments may further promote the extended form populations of the P301L mutant; the dynamic nature of the hinge was shown by the higher values of *R*_2_/*R*_1_ (Figure 2C). The PRI-derived model structure of the wild-type TauF4Δ has moderately exposed PHF6, while the model for the P301L mutant largely exposes the amyloid motif, PHF6. The conformational ensemble switch explains the enhanced aggregation propensity of the P301L mutant. The disrupted inter-segment dynamics by the P301L mutation may facilitate conformational ensembles that differ from those of the wild-type.

This work has shown how the P301L mutation changes the correlated motions among the residues in TauF4Δ, while the mutant has marginal effects on the conformational dynamics in nsec and msec timescales revealed by nuclear spin relaxations, as summarized above. The main finding of this study is that the P301L mutant exclusively changes the transiently forming conformations that expose the amyloid motif PHF6, while the major conformations remain largely unchanged from those of the wild-type. This may explain why the P301L mutation promotes tau aggregation (Figure 8).

Our results support the two-state ensemble model of tau conformations proposed in previous studies [34,35]. In the two-state model, the soluble tau monomer remains in two distinct conformational ensembles [34]. One ensemble is inert to aggregation and remains soluble, whereas the other ensemble contains aggregation-prone conformations. The tau molecules in the aggregation-prone ensemble expose the amyloid motifs PHF6* and PHF6 in extended β-structures, which drive tau to self-assemble [34,35].

FTDP-17 familial mutations [13,73], including the P301L mutation, phosphorylation [74] and polyanion binding [28] may populate the aggregation-prone forms [23,35,36,64]. The P301L mutation reportedly destabilizes the type II β-turn structure of ^301^PGGG^304^ located before the PHF6 motif [23,25,75] (Figure 1), which this work confirmed (Figure 3B). The change in the β-turn structure of ^301^PGGG^304^ by the P301L mutation exposes PHF6 to promote tau self-aggregation [9,22,23]. The PRI-based model structures of the transiently folded wild-type and P301L mutant TauF4Δ obtained herein confirm the mechanism by which the P301L mutation promotes self-aggregation (Figure 7).

The PRE mapping demonstrated that TauF4Δ comprises segments, in each of which the residues show correlated motions (Figure 4). Residues in the β-structures formed in the heparin-induced tau filament [19] constitute segments that display correlated motions (Figure 4). The segments behave as structural elements in fibrillization, although residues in the segments fluctuate significantly on the ns timescale, as evident by their low hNOEs of ~0.25 (Figure 2A).

The segments of the residues in motion have long-range inter-segment correlated motions that make distal residues fluctuate coordinately (Figure 4). The inter-segment correlated motions should define the ensemble structures of the intrinsically flexible TauF4Δ. The PRE map for the P301L mutant TauF4Δ showed that the C-terminal part comprising segments C7 and C8 fluctuate mostly in an anti-correlated manner against the N-terminal C1 segment (Figure 4B), whereas the wild-type protein retains positive correlations for these segments (Figure 4A). The results suggest that the C-terminal part in the P301L mutant moves in the opposite direction when compared with that of wild-type tau (Figure 8). The change in the inter-segment motions likely induces different conformations that transiently occur in the extensive conformational dynamics of TauF4Δ. PRIs detected the transient conformational changes caused by the P301L mutation.

Apparent differences in the PRI maps between the wild-type and P301L mutant TauF4Δ were observed (Figure 5), which suggests that the transiently formed, sparsely populated structures of the P301L mutant have different inter-segment correlated motions when compared with that of the wild-type protein. This is a stark contrast to the PRE observations that showed a limited change in the inter-segment correlation between the C1 and C7–C8 segments. PREs detect all possible conformational dynamics of the protein, whereas PRIs exclusively probe the transiently formed compact structures in which two electron spins are in spatial proximity. The PRE and PRI maps obtained in this study, therefore, suggest that the P301L mutation primarily changes the transiently folded structures, but does not affect most of the conformations of TauF4Δ (Figure 8). The transient structures of the P301L mutant derived from PRI data were shown to expose the amyloid motif PHF6 to prompt self-aggregation, which is consistent with previous studies [17,23,25].

The P301L mutation diminishes the β-structure propensity of the residues in the C6 segment (β3 in the heparin-induced tau filament) and destabilizes the β-turn structure of the linker connecting the C6 and C7 segments (β3 and β4 in the tau filament) (Figure 3). The local changes in the conformation dynamics should disturb the inter-segment correlated motions that connect the entire residues of TauF4Δ, with the aid of the expected hinge motion at the linker connecting the segments C5 and C6 (β2 and β3 in the tau filament) (Figure 2C), should populate the transient aggregation-prone forms in the minor conformation ensemble of the P301L mutant (Figure 8). The molecular dynamics simulation study is anticipated to explore the changes in the correlated motions among the residues in TauF4Δ and will provide clearer insights into how tau mutation changes conformation ensembles.

## 4. Materials and Methods 

### 4.1. TauF4Δ Fragment Preparation

The gene fragment coding TauF4Δ (residues 225–324) was cloned into the expression vector pET28a (Addgene, Watertown, MA, USA). The recombinant plasmid was transformed into *Escherichia coli* (*E. coli*) BL21(DE3) cells (New England Biolabs, Ipswich, MA, USA). The cells were grown in M9 medium with 50 μg/mL kanamycin at 37 °C to an *A*_600_ = 0.8. At this point, protein expression was induced by adding isopropyl β-thiogalactopyranoside (IPTG) to a final concentration of 0.5 mM. After IPTG induction, cells were cultured for a further 5 h. The cells were harvested by centrifugation and resuspended in 60 mL of buffer (50 mM Tris-HCl [pH 8.0], 500 mM NaCl, 2 M urea, 30 mM imidazole). The cell suspension was placed on ice and sonicated, and the cell lysate was centrifuged at 26,740× *g* for 20 min at 4 ℃ to remove cell debris. The supernatant was loaded onto a HisTrap column (GE Healthcare, Chicago, IL, USA) to trap the His_6_-tagged target protein. After washing the column with 30 mL buffer (50 mM Tris-HCl [pH 8.0], 500 mM NaCl, 2 M urea, 30 mM imidazole), the target protein was eluted by the same buffer solution containing 300 mM imidazole.

The collected His_6_-tagged protein sample was extensively dialyzed against 2 L 50 mM sodium phosphate buffer (pH 8.0) containing 2 M urea, 30 mM imidazole and 14 mM β-mercaptoethanol at 4 °C for 6 h. After dialysis, the His_6_-tag was cleaved from the target protein by incubating the sample with His_6_-tagged TEV proteinase (30 μg/mL) overnight at 4 °C. The protein solution was loaded onto a HisTrap column and the flow-through containing the target protein was collected. This sample was loaded onto a HiTrap SP column (GE Healthcare) equilibrated with buffer (50 mM sodium phosphate [pH 8.0], 2 M urea, 30 mM imidazole and 14 mM β-mercaptoethanol). The target protein, TauF4Δ, was obtained by fractionation using a NaCl gradient (0–500 mM) with an AKTA-prime plus HPLC (GE Healthcare). The collected fractions containing TauF4Δ were dialyzed extensively against a 50 mM Tris-HCl (pH 6.8) buffer. The protein was subsequently concentrated by using an Amicon Ultra Centrifugal Filter concentrator (Merck, Darmstadt, Germany). The solution containing purified TauF4Δ was placed in a 90 °C water bath for 5 min to denature contaminated proteinases that may cleave the target protein during long-time storage. The precipitated material following this heating step was removed by centrifugation. The purified TauF4Δ protein was stored at –20 °C.

Site-directed mutagenesis by PCR with KOD FX Neo (Toyobo, Osaka, Japan) was used to prepare TauF4Δ (P301L). Other TauF4Δ mutations (i.e., C291S, C322S, S262C/C291S/C322S, S305C/C291S/C322S, S262C/S305C/C291S/C322S) used in this work for paramagnetic labeling experiments were prepared in the same manner. The mutants were purified according to the procedure described above.

For isotope labeling of TauF4Δ and its mutants, the *E. coli* culture medium contained ^15^NH_4_Cl (0.5 g/L) and/or ^13^C labeled glucose (1 g/L) as sole nitrogen and carbon sources, respectively.

### 4.2. Backbone NMR Resonance Assignments

Backbone resonance assignments of TauF4Δ were obtained using a standard set of three-dimensional triple resonance NMR experiments, including HNCO, HN(CA)CO, CBCA(CO)NH, and HNCACB [76]. All data were collected on a Bruker Avance II NMR spectrometer (Bruker BioSpin, Rheinstetten, Germany) equipped with a cryogenic triple-resonance probe operating at a ^1^H resonance frequency of 700.3 MHz. TauF4Δ was dissolved in a buffer solution containing 50 mM Tris-HCl (pH 6.8), 1 mM DTT, 0.03% NaN_3_ and 6% D_2_O for NMR experiments. All NMR experiments were conducted at 12 °C (285 K).

NMR data were processed by NMRPipe [77]. The resonance assignments were carried out by using the program MagRO [78] on the NMRView platform [79]. Resonance assignments for wild-type TauF4Δ and the P301L mutant were deposited in the Biological Magnetic Resonance Data Bank [80] with accession codes 50129 and 50130, respectively.

### 4.3. NMR Spin Relaxation Experiments

Backbone dynamics for TauF4Δ and its variants were derived from ^15^N nuclear relaxation experiments [47] at a static magnetic field strength of 700 MHz and a temperature of 12 °C (285 K). Each signal intensity in a 2D NMR spectrum was determined by performing nine-point signal intensity averaging with centering at the peak maximum to reduce errors arising from background noise. This averaging was achieved by using a home-written program with each peak center position obtained from the ‘pc’ function in the program SPARKY [81].

Longitudinal relaxation (*R*_1_) experiments were conducted using the following inversion recovery delays: 10.3 (twice), 153.7, 307.4, 461.2, 614.9 (twice), 768.6, 922.3, 1127.2, and 1537.2 ms. Repeat time points were used to determine experimental errors. For the transverse relaxation (*R*_2_) experiments, the durations of the Carr–Purcell Meiboom–Gill (CPMG) loop were 0.0, 32.0 (twice), 80.0, 160.0 (twice), and 320.0 ms. A series of 2D spectra for measuring the *R*_1_ and *R*_2_ rates were collected in an interleaved manner. ^15^N{^1^H} heteronuclear NOE (hNOE) values were measured from pairs of interleaved spectra recorded with (NOE) and without (control) proton saturation during the recycle delay. The duration for ^1^H irradiation was 3 s followed by 2 s to ensure the nuclear spins had returned to the ground state. The recycle delay was 5 s for the control spectrum.

The *R*_1_ and *R*_2_ rates were determined by function fitting to the signal intensities for each ^1^H-^15^N correlation signal by using the modelXY TCL built-in command in NMRPipe [77]. Uncertainty for each relaxation rate was estimated in a Monte Carlo manner using the errors on the peak intensities evaluated from the duplicate datasets. Uncertainties for hNOE values were estimated by determining the root-mean-square deviation value on a spectral region with no signals, which was measured by the command in NMRPipe [77].

Reduced spectral densities, including *J*_eff_(0), *J*(ω_h_) and *J*(ω_N_), were calculated from the above ^15^N relaxation data, including *R*_1_, *R*_2_, and hNOE, using the software suite RELAX version 3.2.3 [49,52,53].

### 4.4. Paramagnetic Relaxation Enhancement (PRE) Experiments

The paramagnetic spin-label, (1-oxyl-2,2,5,5-tetramethyl-δ-3-pyrroline-3-methyl) methanethiosulfonate (MTSL) (Toronto Research Chemicals, Toronto, Canada) was attached to cysteine residues of TauF4Δ and its variants using the following procedure. Prior to spin labeling, 10 mM DTT was added to the protein sample and incubated for 30 min at room temperature to ensure the thiols of cysteines were in the reduced form. The reducing reagent was removed through buffer exchange using a Zeba Spin Desalting Column (Thermo Fisher Scientific, Waltham, MA, USA). Diamagnetic spin labeling with (1-acetoxy-2,2,5,5-tetramethyl-δ-3-pyrroline-3-methyl) methanethiosulfonate (Toronto Research Chemicals) was used for the reference. In labeling, the protein and the labeling reagent were adjusted to a 1:10 ratio, and the sample incubated for 30 min at room temperature. The labeling reagent was removed by buffer exchange using the Zeba Spin Desalting Column.

The transverse relaxation (*R*_2_) rates for backbone amides ^1^H (^1^H^N^) were collected for the paramagnetic- and diamagnetic-labeled proteins to measure the increase in *R*_2_ rates for ^1^H^N^ (^1^H^N^-Γ_2_) caused by the presence of paramagnetism [41,82]. For measuring ^1^H^N^ *R*_2_ rates, four transverse relaxation delays were used: 0.0, 14.0, 28.0, and 42.0 ms. Uncertainties in peak intensities were estimated based on the basal noise level estimated as the root-mean-square deviation on a spectral region with no signals, which was measured by the built-in ‘Estimate Noise’ command in NMRPipe [77]. The experimental errors for *R*_2_ rates were calculated in a Monte Carlo manner using the uncertainties of the peak intensities at each relaxation delay using the modelXY TCL command in NMRPipe [77].

The PRE correlation map was generated based on the ^1^H^N^-Γ_2_ rates for four different paramagnetic labeled proteins (labeled residues are 262, 291, 305, and 322) [40,43,60].

The correlation of the PRE rates (*R*_ik_ and *R*_jk_, residues positions at *i* and *j* with the spin-label at position *k*) between a pair of residues *i* and *j*, *corr*_i,j_, is defined by the differences in the respective rates from their average values (*R*_i0_ and *R*_j0_), as described by the following equations [40,44]:

(1) $$\begin{array}{l} {\mathrm{cov}}_{i,j}=\frac{1}{N}\sum_{k=1}^{N}\left(R_{ik}-R_{i0}\right)\left(R_{jk}-R_{j0}\right)\\ corr_{i,j}={\mathrm{cov}}_{i,j}/\left(\sigma_{i}\sigma_{j}\right) \end{array}$$

where *σ*_i_ and *σ*_j_ are the standard deviations for the PRE rates of residues *i* and *j*, respectively. A pair of residues giving a positive *corr*_i,j_ value fluctuates concertedly in reference to the spin-label. Conversely, residues showing a negative correlation coefficient move in an anti-correlated fashion. The pair of residues with null *corr*_i,j_ values have no correlated motion. 

Only residues giving rise to observable signals for all of the spin-labeled proteins were considered for mapping. A home-written program in MATLAB (MathWorks, Natick, MA, USA) was used for calculating and displaying the correlation map.

### 4.5. Paramagnetic Relaxation Interference (PRI) Experiments

For PRI experiments, two samples with paramagnetic spin labels at two different sites were prepared, i.e., positions 262 and 305, and 291 and 322. 

PRI as the cross-correlated paramagnetic relaxation effect on each ^1^H^N^ from the two paramagnetic labels in the same protein was evaluated by the difference in the ^1^H^N^-Γ_2_ rates (Δ^1^H^N^-Γ_2_) defined as [43,44]:

(2) ΔHN1−Γ2=HN1−Γ2[X(1)+X(2)]−(HN1−Γ2[X(1)]+HN1−Γ2[X(2)])

where the terms ^1^H^N^-Γ_2_[*X*(1)], ^1^H^N^-Γ_2_[*X*(2)] and ^1^H^N^-Γ_2_[*X*(1)+*X*(2)] denote the ^1^H^N^-Γ_2_ rates caused by the single spin-label at residue *X*(1), at residue *X*(2), and the simultaneous spin-labels at residues *X*(1) and *X*(2). 

The PRI correlation map was generated by the same method used to prepare the PRE correlation maps, Equation (1) [40,43,44,45].

## 5. Conclusions

In summary, we have revealed that TauF4Δ comprises segments of residues that undergo correlated motions, and the segments constitute the elementary structural units in the heparin-induced tau fibril. The inter-segment correlated motions probably determine the conformational ensembles of TauF4Δ. Tau is a representative member of IDPs. However, tau is not a polymer physics random coil [83]. Instead, tau has a segmental structure, and each segment behaves as a local structural element, although they are structurally dynamic. The elements play roles similar to those of domains in folded proteins. The P301L mutation altered the transiently folded sparsely populated structures of TauF4Δ by changing the inter-segment correlated motions, and these changes arise from disrupting correlated motions within the segments to induce open conformations that expose the amyloid motif PHF6 (Figure 8). The sparsely populated structures of the P301L mutant explain the elevated aggregation-prone property of the FTDP-17 mutant. 

## Supplementary Materials

Supplementary materials can be found at https://www.mdpi.com/1422-0067/21/11/3920/s1. Figure S1: Correlations between *J*_eff_(0) and *J*(ω_N_) were used to identify residues undergoing chemical exchange for the wild-type (A) and P301L mutant (B) TauF4Δ. Figure S2: Difference in the values of the spectral densities between the wild-type and the P301L mutant TauF4Δ. Figure S3. PRE data for the wild-type TauF4Δ. Figure S4. PRE data for the P301L mutant TauF4Δ. Figure S5. Small-angle X-ray scattering (SAXS) curves for the wild-type (black) and P301L mutant (red) TauF4Δ.

## Abbreviations

NMR	Nuclear magnetic resonance
AD	Alzheimer’s disease
NTF	Neurofibrillary tangle
PHF	Paired helical filament
PTDP-17	Frontotemporal dementia and Parkinsonism linked to chromatin 17
MTSL	(1-oxyl-2,2,5,5,-tetramethyl-d-3-pyrroline-3-methyl) methanethiosulfonate
