# Supplementary material for: Impact of the Hereditary P301L Mutation on the Correlated Conformational Dynamics of Human Tau Protein Revealed by the Paramagnetic Relaxation Enhancement NMR Experiments
Source: Int J Mol Sci. 2020 May 30;21(11):3920. doi: 10.3390/ijms21113920 (PMC7313075; doi:10.3390/ijms21113920)
Supplement: Supplementary file 1 [file ijms-21-03920-s001.pdf]

Supplementary Materials

Impact of the Hereditary P301L Mutation on Correlated Conformational Dynamics of the Human Tau Protein Revealed by Paramagnetic Relaxation Enhancement NMR Experiments

Ryosuke Kawasaki[1] and Shin-ichi Tate[1,2,3,*]

1. Department of Mathematical and Life Sciences, Graduate School of Science, Hiroshima University, 1-3-1 Kagamiyama, Higashi-Hiroshima, Hiroshima 739-8527, Japan; ryosuke-kawasaki@hiroshima-u.ac.jp (R.K.); tate@hiroshima-u.ac.jp (S.T.)
2. Graduate School of the Integrated Sciences for Life, Hiroshima University, 1-3-1 Kagamiyama, Higashi-Hiroshima, Hiroshima 739-8527, Japan; tate@hiroshima-u.ac.jp (S.T.)
3. Research Center for the Mathematics on Chromatin Live Dynamics (RcMcD), Hiroshima University, 1-3-1 Kagamiyama, Higashi-Hiroshima, Hiroshima 739-8526, Japan; tate@hiroshima-u.ac.jp (S.T.)

∗ Correspondence: tate@hiroshima-u.ac.jp: Tel: +81-82-424-7387

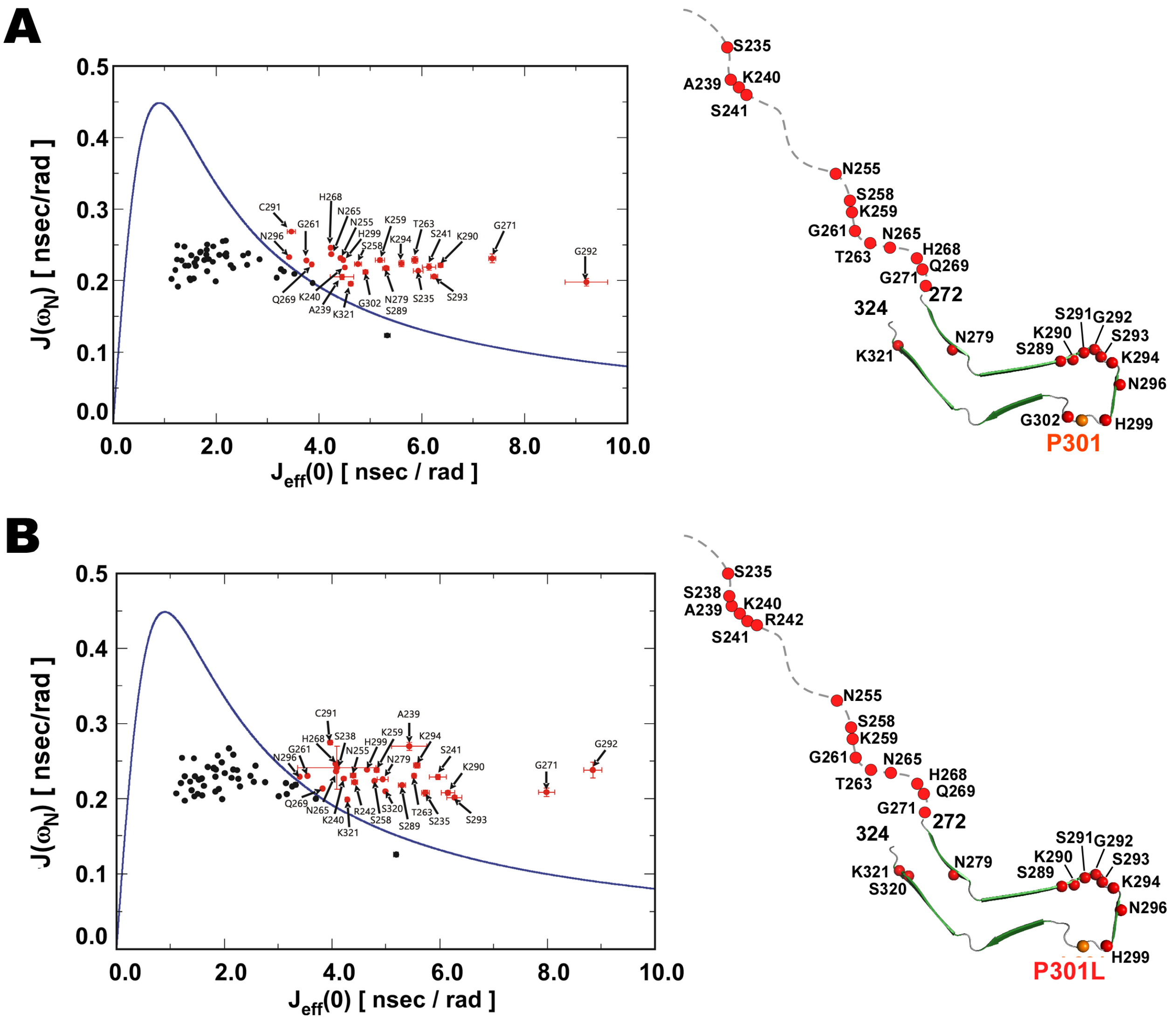

Figure S1. Correlations between $J_{\mathrm{eff}}(0)$ and $J(\omega_N)$ were used to identify residues undergoing chemical exchange for the wild-type (A) and P301L mutant (B) TauF4Δ. Data points (red) that elevated $J_{\mathrm{eff}}(0)$ from the theoretical correlation curve (blue line), eq. (1), represent amide bonds of residues that have significant chemical exchange contributions to the ^{15}N nuclear spin relaxation processes, indicating that these residues undergo backbone conformational fluctuations on the μsec – msec time regime [1].

Assuming that there are no local amide bond motions, backbone amide ^{15}N nuclear spin relaxation is dominated by the overall rotational correlation time. Under this condition, the spectral densities $J_{\mathrm{eff}}(0)$ and $J(\omega_N)$ are correlated by the following equation:

$$J(\omega_N) = \frac{J(0)}{1 + 6.25(\omega_N J(0))^2} \qquad (1)$$

where, ω_N is the ^{15}N Larmor frequency. A graphical representation of eq. (1) is shown by the blue line.

Outlier residues are marked by red circles on the heparin-induced tau filament structure (PDB ID: 6QJH) [2]. The grey dotted line represents the TauF4Δ fragment that was not observed in the cryo-electron microscopy structure of the tau filament.

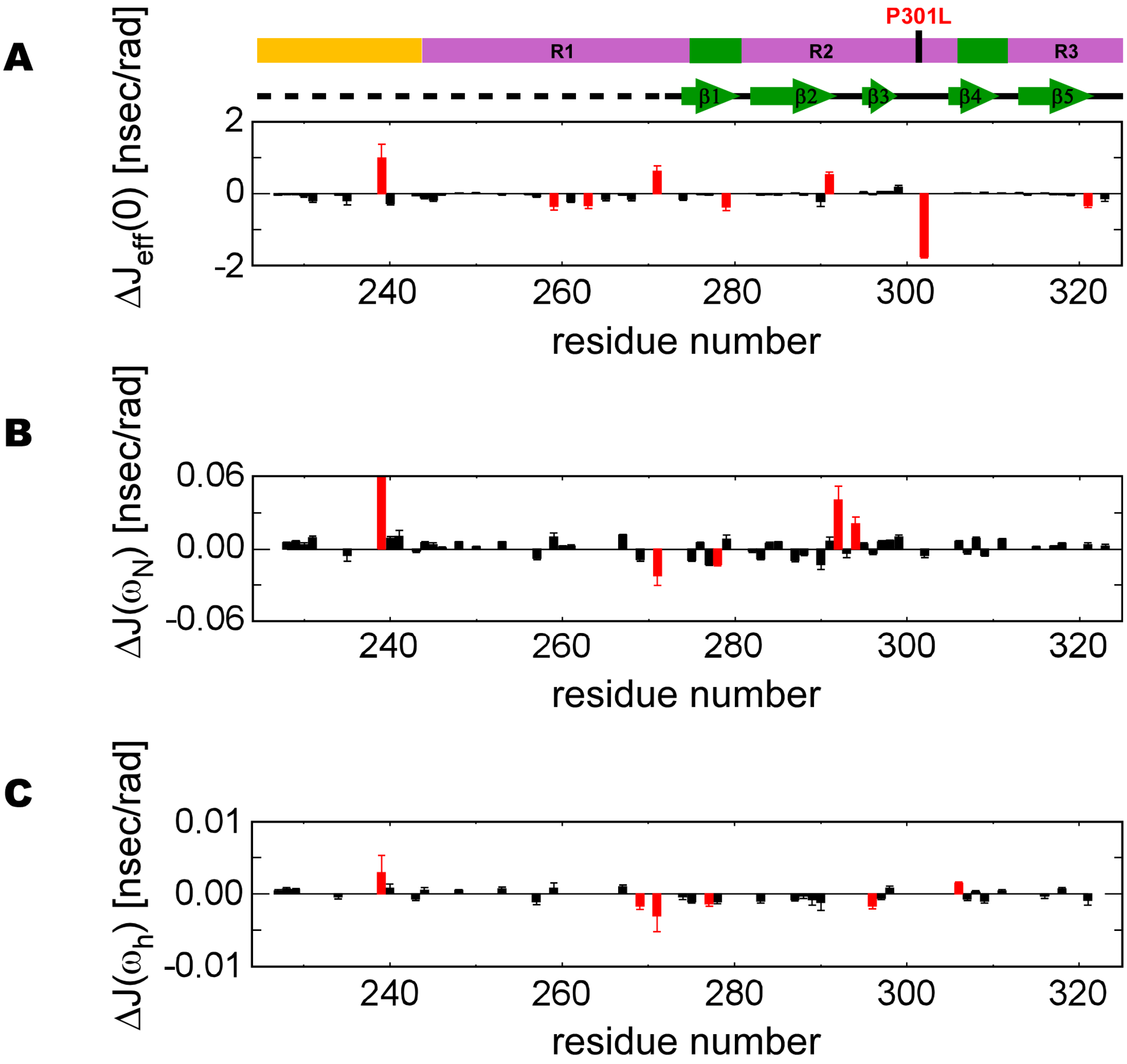

Figure S2. Difference in the values of the spectral densities between the wild-type and the P301L mutant TauF4Δ. (A) $J_{eff}(0)$, (B) $J(\omega_N)$, and (C) $J(\omega_h)$. The differences were calculated as J(P301L) – J(wild-type), where J represents a spectral density. The bars in red show the difference is significantly greater than the error.

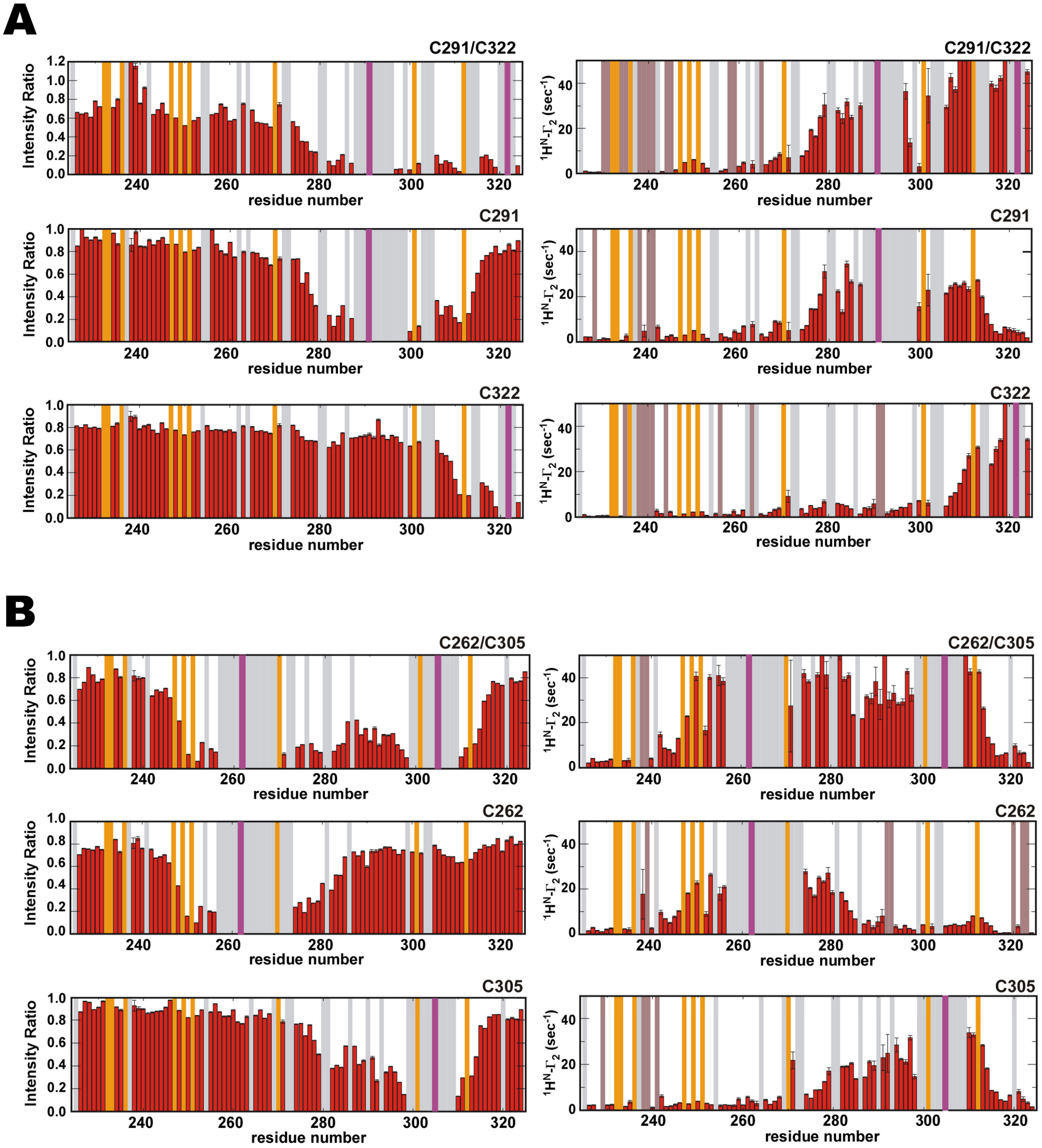

Figure S3. PRE data for the wild-type TauF4Δ. (A) PRE data for the wild-type labeled at C291 or C322, and PRI data for the wild-type labeled at C291 and C322 (red bars). Left panels show the intensity ratios of the ^{1}H-^{15}N HSQC signals measured for the paramagnetic labeled protein in reference to the diamagnetic labeled reference protein. Right panels display the PRE rates, $^{1}H^{N}$-Γ_2, measured for the proteins labeled at the position(s) on the right top of each graph. The bars in orange represent the positions of proline residues that do not give rise to signals in ^{1}H-^{15}N HSQC spectra. The dark pink bars indicate the position(s) for the spin-labeled residue(s). The gray bars represent residues that did not give rise to any resolved signals used for PRE analyses.

(B) The same PRE data set for the proteins singly labeled at C262 or C305 and the double-labeled protein at C262 and C305 (red bars).

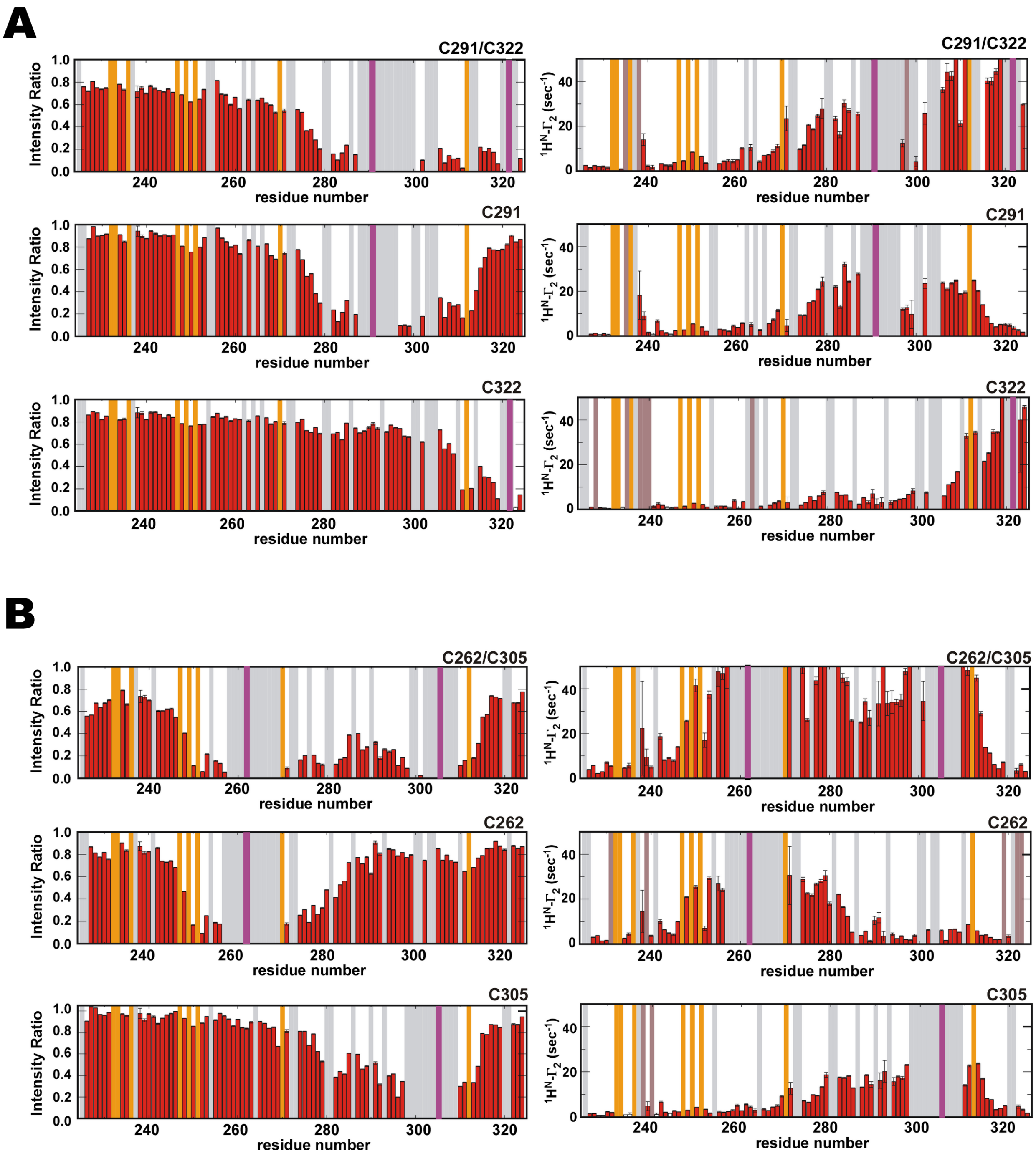

Figure S4. PRE data for the P301L mutant TauF4Δ. (A) PRE data set for the proteins singly labeled at C291 or C322, and the protein double-labeled at C291 and C322 (red bars). Left panels display the intensity ratios of the protein labeled with paramagnetic spin(s) in reference to the diamagnetic labeled protein. Right panels show the corresponding PRE rates, $^1H^N$-Γ_2, to the data presented on the left. (B) PRE data for proteins labeled at C262 or C305, and the double-labeled protein at C262 and C305 (red bars). The definitions of the other colored bars are the same as those used in Figure S3.

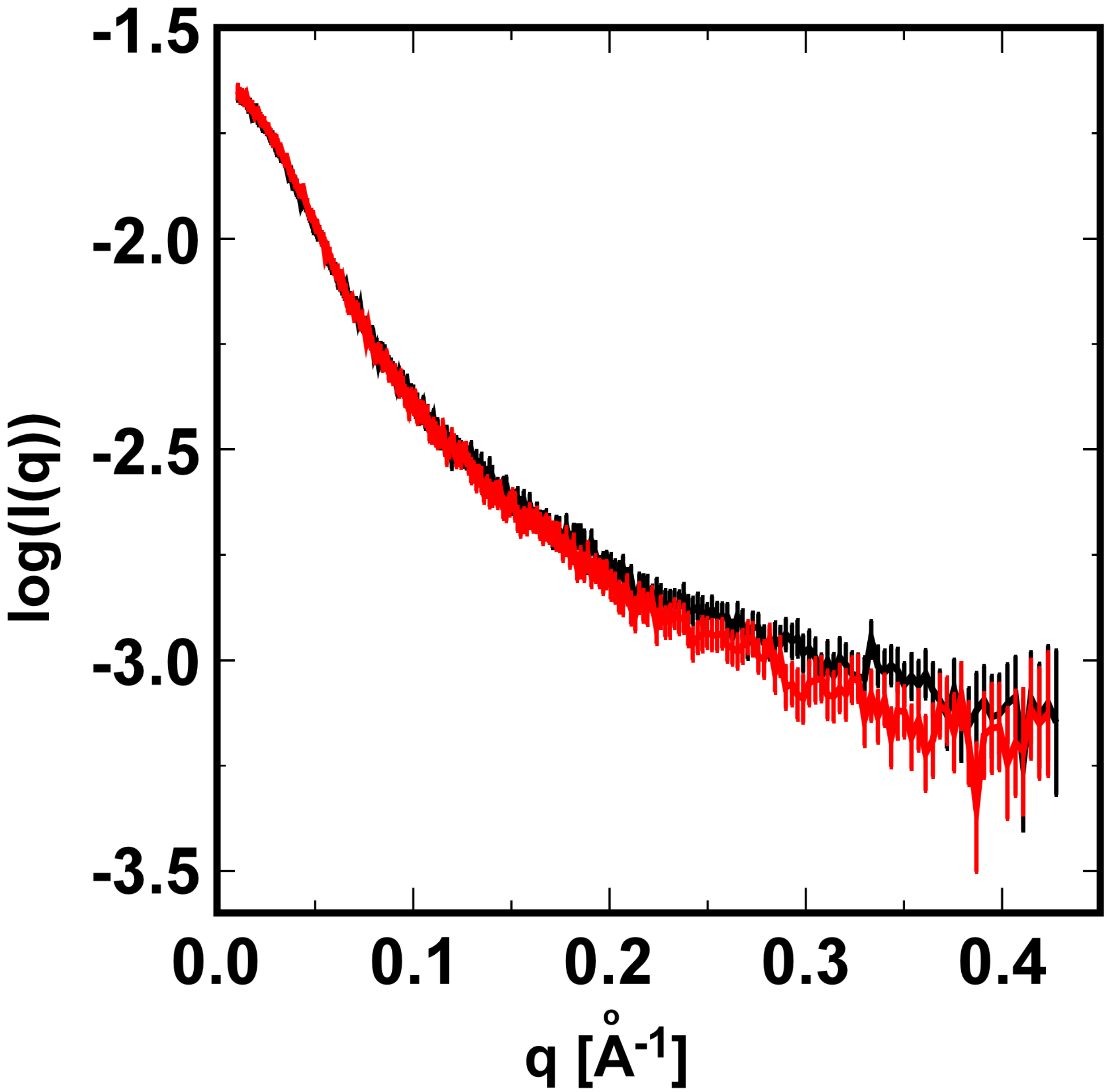

Figure S5. Small-angle X-ray scattering (SAXS) curves for the wild-type (black) and P301L mutant (red) TauF4Δ. The scattering data within the low-resolution region (q < 0.1 $Å^{-1}$) represent the average square distance of each scatter from the center of the molecule giving X-ray scattering [3]. Therefore, the scattering profile in the low-resolution region is proportional to the apparent Stokes radii of the molecule or the end-end distance of the polypeptide chain [3]. The similarity of the SAXS profiles between the wild-type and P301L mutant in the low-resolution region demonstrates that these two proteins have similar molecular sizes in solution.

All SAXS samples were purified using size exclusion chromatography (SEC) to remove aggregated components prior to data collection. All SEC-SAXS experiments were carried out on the BL23A beamline at the National Radiation Research Center (NSRRC, Hsinchu, Taiwan). The protein concentrations were adjusted to 15 mg/mL in a buffer solution composed of 50 mM Tris-HCl (pH 6.8) and 10 mM β-mercaptoethanol. The data were collected with a momentum transfer q range of 0.006–0.439 $Å^{-1}$, which corresponds to an X-ray wavelength of λ = 0.83 Å, 15 keV. The beam geometry was 0.5 × 0.5 mm^2. The sample temperature was set to

15 °C using an oil-bath circulation system. The solvent signal was subtracted from the collected scattering data for the protein solution by using a home-written program.

References

1. Křížová, H.; Žídek, L.; Stone, M.; Novotny, M.; Sklenář, V. Temperature-dependent spectral density analysis applied to monitoring backbone dynamics of major urinary protein-I complexed with the pheromone 2-sec-butyl-4,5-dihydrothiazole*. *Journal of Biomolecular NMR* **2004**, *28*, 369-384, doi:10.1023/B:JNMR.0000015404.61574.65.
2. Zhang, W.; Falcon, B.; Murzin, A.G.; Fan, J.; Crowther, R.A.; Goedert, M.; Scheres, S.H. Heparin-induced tau filaments are polymorphic and differ from those in Alzheimer's and Pick's diseases. *Elife* **2019**, *8*, doi:10.7554/eLife.43584.
3. Putnam, C.D.; Hammel, M.; Hura, G.L.; Tainer, J.A. X-ray solution scattering (SAXS) combined with crystallography and computation: defining accurate macromolecular structures, conformations and assemblies in solution. *Q Rev Biophys* **2007**, *40*, 191-285, doi:10.1017/S0033583507004635.
